# Supplementary figures and images for: Experiences and lessons learned from community-engaged recruitment for the South Asian breast cancer study in New Jersey during the COVID-19 pandemic
Source: PLoS One. 2023 Nov 13;18(11):e0294170. doi: 10.1371/journal.pone.0294170 (PMC10642833; doi:10.1371/journal.pone.0294170)

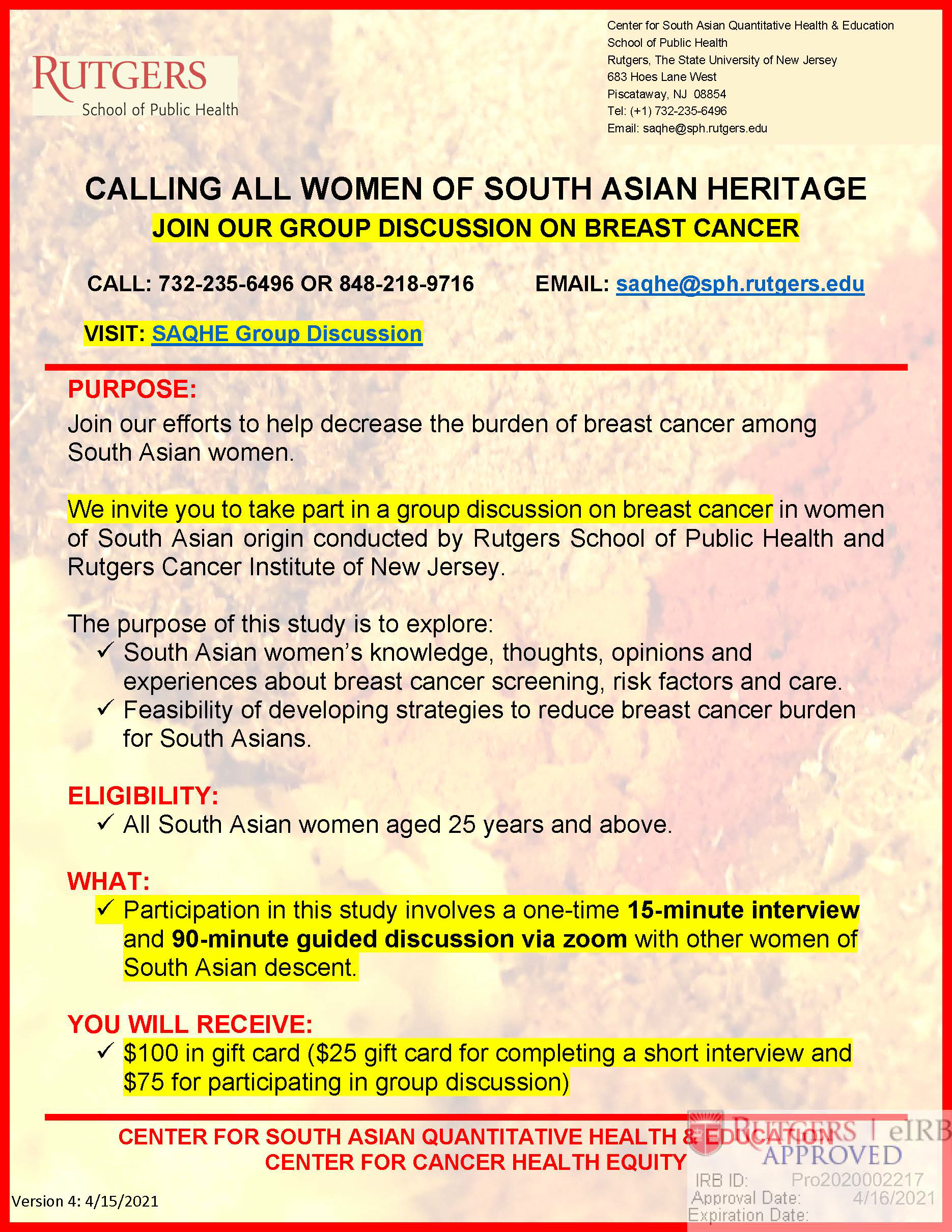

Supplement: S1 Fig — The watermark shows heaps of spices commonly used South Asian cuisines. The yellow colors represent turmeric, a commonly used spice. The red color in the border and horizontal lines represent powdered chili, a commonly used spice, and also represent henna and vermillion, which are commonly used for cosmetic needs in the South Asian culture. The phrase “VISIT: SAQHE Group Discussion” gave a link to a Rutgers-approved Qualtrics survey page where interested individuals could provide their contact information (phone number and email address) so that study staff could contact them. (TIF) [file pone.0294170.s001.tif]

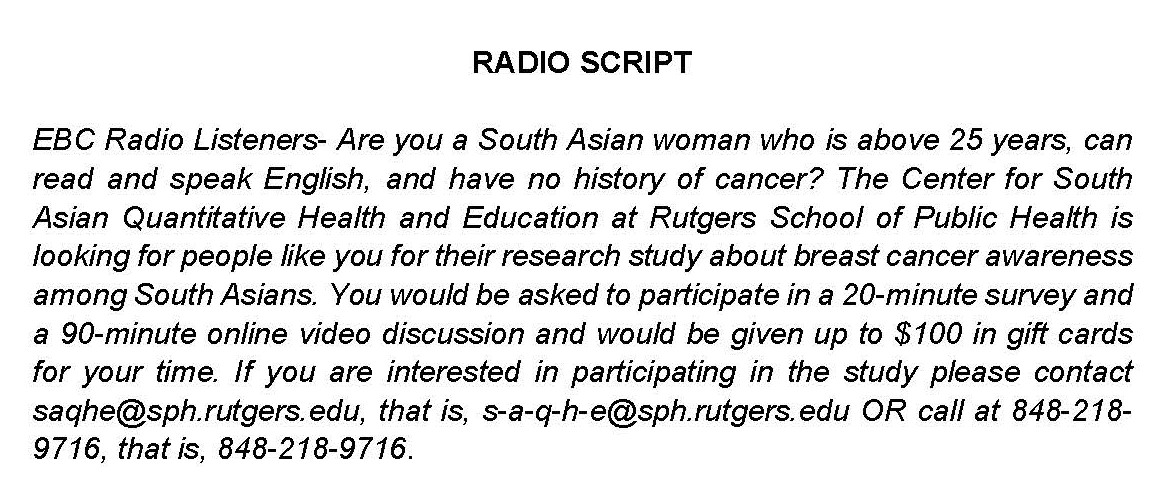

Supplement: S2 Fig — This IRB-approved script was used to disseminate the study information through the community radio. (TIF) [file pone.0294170.s002.tif]
