# Supplementary material for: Experiences and lessons learned from community-engaged recruitment for the South Asian breast cancer study in New Jersey during the COVID-19 pandemic
Source: PLoS One. 2023 Nov 13;18(11):e0294170. doi: 10.1371/journal.pone.0294170 (PMC10642833; doi:10.1371/journal.pone.0294170)
Supplement: Attachment — Submitted filename: PlosOne -Lessons learnt from community based recruitment --Reviewer comment.docx [file pone.0294170.s003.docx]

**Manuscript Information**

| Manuscript Number: | PONE-D-23-05763 |
| --- | --- |
| Manuscript Title: | Lessons learned from community-based recruitment for the south Asian breast cancer study during the COVID-19 pandemic |
| Date Sent To Reviewer: | **23^rd^ April, 2023** |

**Evaluation Report**

| Title | **Main title:**   - There is no indication of where the study was conducted; the way it is stated it would imply that the study was conducted in South Asia - Authors should modify the title to give idea of the study location |
| --- | --- |
| Abstract | **Methods:** Authors should state the category of people recruited and where (location) the recruitment covered   - The study design, number of participants should also be mentioned   **Results:**   - On line 30, authors stated that they wanted to “gain insights into breast cancer-related health attitudes of South Asian American”, however there is no single figure in the result regarding breast cancer health related attitudes of the women; the result mainly focused on awareness of the women on the study and their sources of information. - Authors should re-write the result section in line with the aim of the study, otherwise they will need to modify the study title in line with what they presented (i.e. awareness on the breast cancer study on sources of information)   **Conclusion:** Does not appear to be based on your objectives; kindly modify it to capture your objectives  **Key words**:   - Authors did not provide any keyword; kindly provide at least 3-5 keywords |
| Introduction | 1. There is need for authors to fragment the 1^st^ paragraph (line 54-85) into different paragraphs as new ideas are introduced 2. Authors did not provide information regarding breast cancer-related health attitudes attitude of women as reported in other studies; this will give insight into the burden of the problem, since it is part of the objectives of the study 3. Rationale/aim of the study should be taken towards the end of the introduction (preferable last paragraph) 4. There is need to show the various nuanced approaches used in different parts of the globe as reported in similar studies |
| Methodology | **Study area:**   - Authors should begin this section with description of the study area   **Study population:**   - Authors need to state the study population before describing the eligibility criteria   **Study design**:   - Kindly state the study design used   **Instrument of data collection**:   - Authors did not describe the instrument of data collection; they should kindly state and describe the instrument used for the data collection   **Data source and collection:**   - Authors said they used FGD, however, there is no description of the FGD that was conducted, what categories of participants constituted each focus group, were the groups homogenous? Etc Authors need to provide more information on these issues   **Recruitment method:**   - In line 133, the authors said snowball sampling was used to recruit participants, however, in line 134/135 they said passive sampling….was used; there is need for clarification. - If snowball sampling was used, what was the rationale behind that?   **Fig 1. Timeline of community engagement strategies**.   - No figure shown in the manuscript; what authors presented as figure (last page) looks more like a table rather than figure   **Community organizations**   - The detail description of the organizations given by authors is not necessary as they are not the focus of the study   **Community-focused health education events**   - This sub-heading gives the impression that the study was an intervention study (educational intervention); authors may need to concentrate on describing methods related to attaining the objectives of their study   **Sample size**:   - Authors said they sought to recruit 18 to 32 eligible participants to conduct 3 to 4 focus groups; authors should state the actual number of participants recruited and the number of FGD groups since the study has been conducted already - For a qualitative research that adopted FGD as the only method of data collection, optimal number of FGDs to be conducted is determined by data saturation rather than a predetermined number set by the researcher.   **Statistical analysis**:   - The description given by authors gives the impression that quantitative data analysis was done. If that was the case, the sample size is too small and there would be need to show the formula that was used to calculate the sample size - Authors did not describe how the FGD was analysed; was it narrative synthesis or it was using software such Nvivo etc? |
| Results | **Result:**   - Most part of the result concentrated on describing participants’ characteristics rather than providing information on the objectives of the study - No result for the FGD?   **Lessons learned**   - From the list of lessons learned, it is clear the research focused communication/community engagement strategies rather than breast cancer-related attitude of women |
| Discussion |  |
| Conclusion/recommendations | - Authors should provide conclusion and recommendations |
| Abbreviations | Provide meanings for some of the abbreviations used in the main text |
| Bibliography/References | Reference list (No. 2, 25): Correct authors’ name in line with Vancouver referencing style  Reference list (No. 6,16,18,28,33): update page number (or provide web address/access date)  Reference list (No. 24): Provide place of publication  Reference list (No. 19,20,21,22,26): provide date article was accessed from the web source  Reference list (No. 38): web address provided is not accessible (remark: “page not found”) |
| Others | Nil |
| Final Note | The manuscript may be considered for publication after effecting corrections. |

Please rate the following: (1 = Excellent) (2 = Good) (3 = Fair) (4 = poor)

| Originality | **2** |
| --- | --- |
| Contribution To The Field | **2** |
| Technical Quality | **3** |
| Clarity Of Presentation | **2** |
| Depth Of Research | 3 |

**Recommendations**

Kindly mark with an X

| Accept As It Is |  |
| --- | --- |
| Requires Minor Corrections |  |
| Requires Moderate Revision |  |
| Requires Major Revision | **X** |
| Not Suitable for Further Processing (Please be clear and specific) |  |
